# Clinical Audit: The Quality of Medical Recommendations

**DOI:** 10.1192/bjo.2026.11800

**Published:** 2026-06-30

**Authors:** Abdulrahman Nafiu, Esther Loganathan, Michael Ventress

**Affiliations:** South West Yorkshire Partnership NHS Foundation Trust, Wakefield, United Kingdom

## Abstract

**Aims:**

**Background**

The decision to detain an individual under mental health legislation represents one of the most profound and important decisions in psychiatry. It is a decision that must be grounded in a meticulous and clearly documented clinical justification as to why someone should be deprived of their liberty. Section 2 and section 3 medical recommendation statutory forms state that reasons should be given to explain how symptoms and behaviour lead you to an opinion. These forms are scrutinised and anecdotal evidence suggested the need for an improvement in documentation quality.

**Aim**

The aim of this audit was to compare current practice in South West Yorkshire Partnership Teaching NHS Foundation Trust to statutory guidance in the Mental Health Act 1983: Code of Practice and to what the statutory forms state should be included in a medical recommendation.

**Methods:**

Ethical Consideration and Project Advice

We held a preliminary meeting with the Head of Research, in the Trust Clinical Research Team - this meeting was held for project advice and to discuss ethical considerations.

Key points discussed:

1. The project would involve no patient contact.

2. It will not alter patient care of the patients whose medical recommendations are being assessed for the audit.

3. It was classified as a significant safety project from the perspective of the Head of Research and Information Governance Manager.

4. Head of Research provided an exemption record to authorize access to the necessary patient records.

**Data Sources**

- Sample of 50 medical recommendations - from January 2025 onwards (until reached 50).
- For admission for assessment (under section 2 MHA 1983), or for admission for treatment (under section 3 MHA 1983), both single and joint recommendations in the Wakefield area.
- Mental Health Act Office – sent a list of NHS numbers via email.

**Data Collection**

A Microsoft Excel spreadsheet was designed as the primary data collection tool to streamline and standardize the process. The form included fields aligned with the key parameters outlined in the Mental Health Act 1983: Code of Practice and statutory forms. Data was collected from the sample, between August and September 2025.

**Data Analysis**

Anonymous data was added to the Excel spreadsheet, and this was reviewed by two reviewers / assessors (one of which was section 12 approved). On occasions where the assessors were unable to agree, the recommendation was then sent to another reviewer (who was section 12 approved).

**Audit Standards - Compliance of 100% Expected**

1. Did the doctor state that the patient was suffering from a mental disorder?

2. Did the doctor describe symptoms of the mental disorder?

3. Did the doctor cover nature OR degree sufficiently?

4. If necessary for the patient’s own health, were adequate reasons given?

5. If necessary for the patient’s own safety, were adequate reasons given?

6. If necessary for the protection of other persons, were adequate reasons given?

7. Did the doctor explain why community treatment was not available and appropriate?

8. Did the doctor explain why informal admission was not appropriate?

**Results:**

The below results were found in terms of compliance with standards set.

1. Did the doctor state that the patient was suffering from a mental disorder? - 100%

2. Did the doctor describe symptoms of the mental disorder? - 100%

3. Did the doctor cover nature OR degree sufficiently? - 100%

4. If necessary for the patient’s own health, were adequate reasons given?-100%

5. If necessary for the patient’s own safety, were adequate reasons given? - 62%

6. If necessary for the protection of other persons, were adequate reasons given? - 81%

7. Did the doctor explain why community treatment was not available and appropriate? - 90%

8. Did the doctor explain why informal admission was not appropriate? - 100%

**Conclusion:**

**Key Successes**

We found 100% compliance with stating the patient was suffering from a mental disorder, describing symptoms of the mental disorder, covering nature or degree, giving adequate reasons for why detention necessary for patient’s own health and explaining why informal admission was not appropriate.

**Key Concerns / Areas for Improvement**

We found partial compliance for giving adequate reasons for why detention was necessary for the protection of other persons and why community treatment was not available and appropriate. There was poor compliance for giving adequate reasons why it is necessary for the patient’s own safety.

These statutory forms are the basis on which individuals are deprived of their liberty. There are elements of the form which are mandatory and despite the vast majority of medical recommendations being made by those who are section 12 approved, for at least one of the statutory criteria (why it was deemed necessary for the patient’s own safety), only 62% of these doctors provided the required evidence to support that element of their opinion.

**Limitations of the Project**

- The samples provided by the Mental Health Act office only had section 3 medical recommendations and single recommendations which may have skewed the results.
- The data collection was based on the subjective opinion of assessors – but the forms were reviewed by two assessors and discussed with third assessor if the two assessors could not agree.
- The data was collected over a five-month period, and we considered if a longer period of data collection may have given a more longitudinal view.

**Key Actions to be Implemented**

- We shared the results locally with doctors in the Trust and plan to share the results with the Trust Mental Health Act Committee.
- We have proposed a system of giving feedback to doctors about their medical recommendations and the introduction of an agreed template to improve the quality of these as well.
- We plan to re-audit following implementation of changes after three to six months.

